# Structural Heart Interventions in Patients with Left Ventricular Assist Devices

**DOI:** 10.31083/RCM27964

**Published:** 2025-04-24

**Authors:** Puvi Seshiah, Eugene Chung, Santiago Garcia, Joseph Choo, Robert Dowling, Geoffrey Answini, Dean Kereiakes, Gregory Egnaczyk

**Affiliations:** ^1^The Christ Hospital Heath Network, The Heart & Vascular Institute, Cincinnati, OH 45219, USA; ^2^Department of Cardiology, Charleston Area Medical Center/Vandalia Health, Charleston, WV 25304, USA

**Keywords:** left ventricular assist device, transcatheter interventions, valvular heart disease

## Abstract

Left ventricular assist devices (LVADs) have changed the landscape for patients with advanced heart failure (HF). With advances in pump design and management, patients with LVADs are living longer with improved quality of life despite having more comorbidities and complex structural heart disease. As such, HF cardiologists and surgeons collaborate more frequently with structural heart interventionalists to address the complex problems of patients with LVADs who present at different points of failure in their circuits. Unlike heart transplants and total artificial heart recipients, the native heart and its components must function to maintain successful circulatory support from these assist devices. Multiple points of potential failure of the native heart and the LVAD circuit exist that can result in significant morbidity and mortality. These include regurgitant valve lesions, interatrial shunts, outflow cannula obstruction, and pump thrombosis. Transcatheter interventions can be applied and tailored specifically to the anatomy of the individual in these situations to improve the lives and outcomes of our LVAD patients. This review provides a comprehensive approach for diagnosing and treating structural heart disease associated with patients who have LVADs, focusing on multidisciplinary collaboration and individualized interventional strategies.

## 1. Introduction

**Fig. 1.**
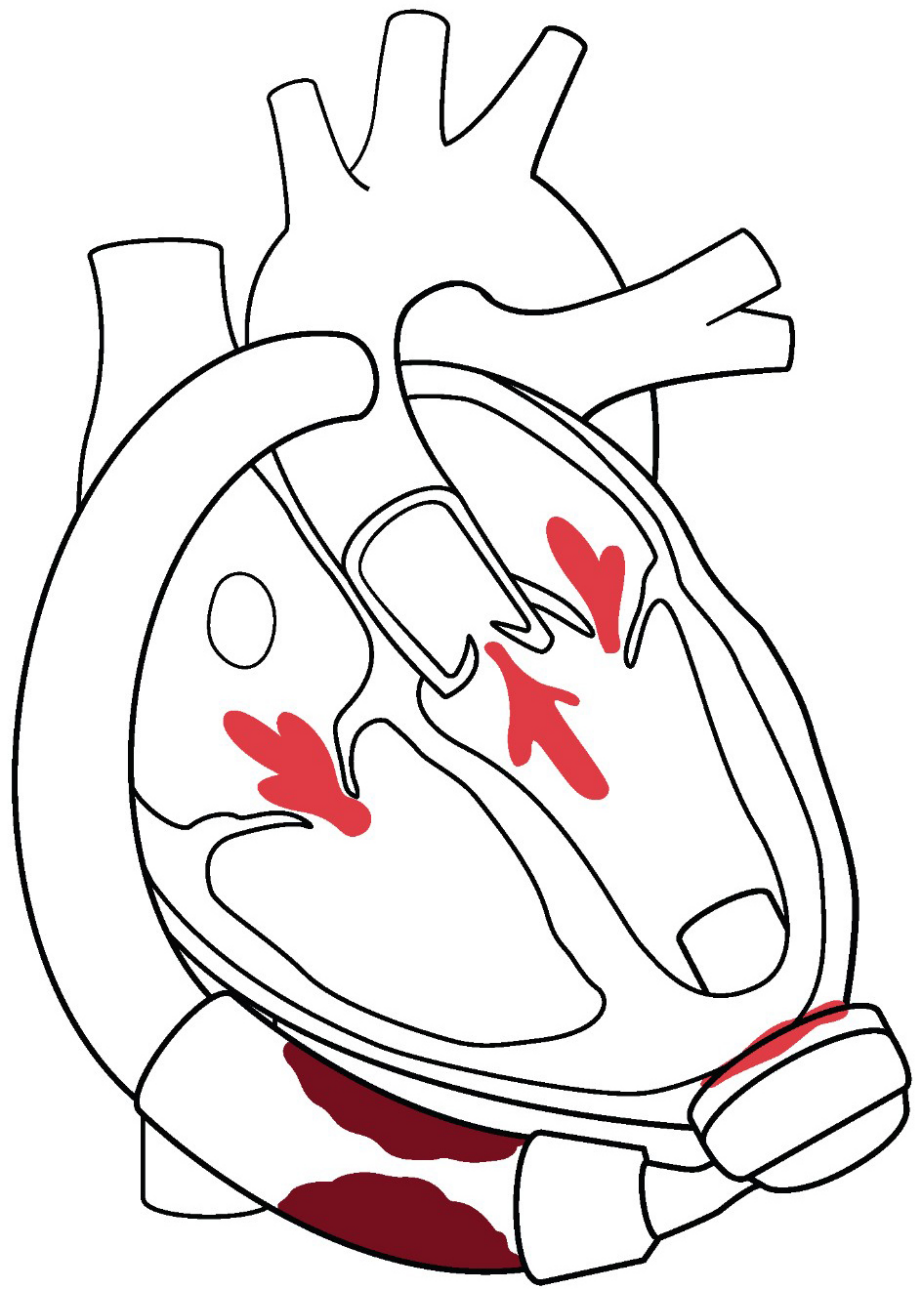
**Overview of structural heart interventions in LVAD patients**. Multiple valve lesions can arise post LVAD that can be addressed with transcatheter valvular therapies. Aortic regurgitation can be treated with TAVR. Mitral and tricuspid regurgitation can be treated both by transcatheter edge to edge repair, as well as TMVR/TTVR strategies. PFO and iatrogenic ASD can complicate post LVAD management, typically by right to left shunting resulting in refractory hypoxemia. Post LVAD complications of pump thrombosis and outflow cannula obstruction can be treated in the catheterization laboratory. De-activation of the LVAD for patients with recovery require a hybrid procedure achieved in part by transcatheter delivery of closure devices inside the outflow cannula. LVAD, left ventricular assist devices; TAVR, transcatheter aortic valve replacement; TMVR, transcatheter mitral valve replacement; TTVR, transcatheter tricuspid valve replacement; PFO, patent foramen ovale; ASD, atrial septal defect.

Left ventricular assist device (LVAD) have changed the landscape for patients with advanced heart failure (HF). Evolution in LVAD pump design and improvements in post-surgical and medical management has led to improved survival for LVAD patients with reported 5-year survival for HeartMate 3 (HM3, Abbott Laboratories, Minneapolis, MN, USA) LVAD at 58.4% [1]. With longer periods of LVAD therapy, there are more opportunities for a variety of circulatory abnormalities to develop leading to impaired hemodynamics, recurrent heart failure hospitalizations and inadequate LVAD flows. Unlike heart transplant and total artificial heart recipients, the native heart and its components need to function to maintain the successful circulatory support from these assist devices. Multiple points of potential failure of the native heart and the LVAD circuit can result in significant morbidity and mortality (Fig. 1). These include regurgitant valve lesions, interatrial shunts, outflow cannula obstruction and pump thrombosis. Transcatheter interventions can be applied and tailored specifically to the anatomy of the individual in these situations to improve the lives and outcomes of our LVAD patients.

Development of aortic regurgitation (AR) after LVAD implantation is common, with at least moderate or severe aortic regurgitation occurring in up to 25% in the first year for early generation continuous flow devices [2]. The incidence of significant AR is reduced with the HM3 LVAD, down to 8% at 2 years, however the longer patients are on the support the prevalence continues to increase. Both mitral (MR) and tricuspid (TR) regurgitation are problematic in the management of these patients, particularly in light of the concomitant right ventricular dysfunction and renal failure. In an INTERMACS analysis, 18.8% of LVAD patients developed moderate or more MR within the first 3 months of LVAD implant and was associated with worse hemodynamic and clinical outcomes [3]. In a EUROMACS analysis, 10% of patients with no significant baseline TR developed mod or more TR immediately after LVAD surgery. LVAD patients with residual moderate or more TR had worse long-term mortality [4]. As patient time on LVAD support increases, outflow graft obstruction due to extrinsic compression has been an increasing recognized complication. In a single center study of 347 patients, incidence of outflow cannulas stenosis requiring treatment was 4.9%, with incidence rising with passing time [5]. Structural abnormalities in the native heart or LVAD pump or circuit are usually not amenable to surgery due to high risk, and transcatheter approaches are the only feasible methods. The lack of large-scale studies to guide management, and reliance on case reports and anecdotal events truly impedes evidence-based care. However, more robust reporting of various management strategies should form a mosaic of options to better inform the LVAD physician community.

These patients with their complex anatomy and hemodynamics require a multidisciplinary heart team approach. HF cardiologists and surgeons are collaborating more frequently with structural heart interventionalists to address the complex problems of LVAD patients who present at different points of failure in their circuit. The structural interventional cardiologists and cardiac surgeons are facile with the growing armamentarium of percutaneous options. The HF cardiologists have taken care of the LVAD patients as primary care physicians and readily recognize when there is a change in a patient symptoms and functional capacity. Finally, advanced cardiac imagers can help decipher the complex anatomy and inform procedural strategy. Commonly, multiple valvular lesions occur in on patient and the impact and timing of transcatheter intervention on single and multiple valves needs to be carefully considered. Right ventricular (RV) dysfunction often co-exists in LVAD patients with progressive valvular disease and requires careful assessment with advanced cardiac imaging and invasive hemodynamics. The dialogue across all the heart team members crystallizes into an expert care plan individualized to each patient. In most cases the recommendation of the heart team represents off-label uses of devices. Larger scales prospective studies are needed to validate the efficacy of these interventions. This review provides a comprehensive approach for the diagnosis and treatment of structural heart disease associated with patients who have LVADs, with a focus on multidisciplinary collaboration and individualized interventional strategies.

## 2. Aortic Regurgitation

### 2.1 Etiology in LVAD Patients

**Fig. 2.**
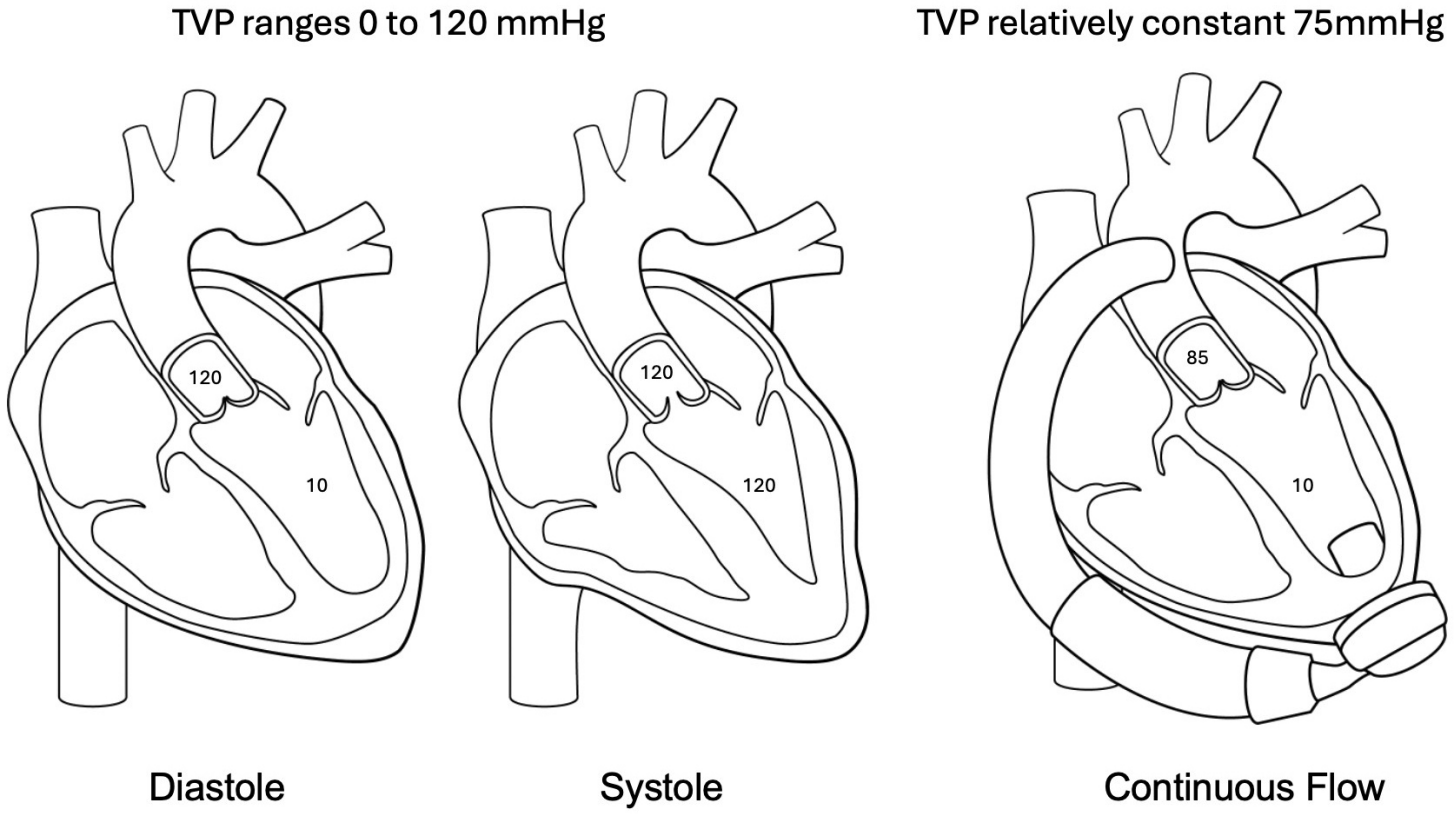
**Pathophysiology of aortic regurgitation post LVAD**. In native circulation the transvalvular pressure (TVP) ranges from zero to peak systolic aortic pressure allowing a time of minimal stress on the aortic valve leaflets. In continuous flow circulation the TVP remains relatively constant thus creating an increased hemodynamic stress on valve leaflets contributing to the development of aortic regurgitation (AR) post LVAD.

Aortic blood flow dynamics play a key role in the development of AR related to continuous flow LVAD. The LVAD extracts blood from the left ventricle and directs the flow to the ascending aorta at a much higher velocity (compared to the normal aorta) due to the smaller size of the conduit compared to the aorta. This leads to higher aortic root pressure while simultaneously decreasing left ventricular (LV) pressure creating an elevated and continuous transvalvular pressure gradient (Fig. 2). In addition, shear stress in the aorta from continuous flow results in abnormal aorta wall morphology contributing to dilatation and AR. A third potential mechanism of AR related to LVAD is commissural fusion of the aortic valve [6]. Reduced excursion of the aortic valve from decreased LV ejection promotes valve thickening, reduced valve pliability and fusion with subsequent degeneration accelerated by valve trauma from high-velocity blood flow.

The unique hemodynamic pressure on the aortic valve in non-pulsatile, continuous flow LVADs can lead to progressive aortic regurgitation over time. In the Momentum trial, the development of moderate or worse aortic regurgitation at 2 years was 18% for the HeartMate 2 (HM2) device and 8% for the HM3 device [7]. Risk factors for development of significant aortic regurgitation included female sex (HR 3.65) and increased age (HR 1.49 for every 10 years). A single center analysis from Duke had similar incidence of AR and corroborated female sex as a risk factor for progression [8]. Surgical factors that contribute to de novo aortic regurgitation post LVAD include angulation of the outflow cannula and position in the ascending aorta [9]. Post LVAD factors that lead to progressive and de novo aortic regurgitation include systemic arterial hypertension and persistent aortic valve closure.

### 2.2 Considerations at Time of Implant

Due to this progressive nature and significant impact on effective LVAD circulatory support, aortic regurgitation should be treated aggressively at time of LVAD implant. The 2023 ISHLT guidelines [10] recommend that (1) More than mild aortic regurgitation should be addressed at the time of LVAD implant. Aortic valve replacement using a biologic valve should be performed, if necessary and (2) Aortic valve closure techniques may be considered to address more than mild aortic regurgitation in selected patients.

At time of surgery three options may be considered to manage aortic regurgitation (1) Aortic valve repair (2) Aortic valve replacement (AVR) with a bioprosthetic valve (3) Oversewing the valve closed. Mechanical AVR is not recommended. The most performed procedure for aortic regurgitation is a central aortic valve cusp approximation (“Park stitch”). The 3-year outcomes of this procedure appear promising [11]. Suture closure technique of the native aortic valve with felt strips and the use of a circular patch of glutaraldehyde-treated bovine pericardium, sewn circumferentially to the aortic valve (AV) annulus permanently closing the left ventricular outflow tract have been performed with a low rate of AR recurrence. However, this leaves the patient completely dependent on the LVAD for circulation, and adverse events such as pump thrombosis or malfunction could be devastating. This technique should not be used when myocardial recovery is possible or expected. An INTERMACS database analysis of concomitant aortic valve procedures at time of LVAD compared AV repair [n = 125], closure [n = 95], and replacement [n = 85] reported increased mortality associated with complete oversewing of the valve, with most deaths occurring early after the procedure [12]. AV repair or replacement was associated with better one-year survival than AV closure. One-year survival in this analysis was as follows: 81% for patients who did not undergo an AV procedure, 79% for patients who underwent an AV repair, 72% for patients with an AV replacement, and 64% for those with AV closure.

Aortic valve replacement with a biological valve may be considered, however rapid bioprosthetic degeneration compared to non- LVAD patients and increased thrombosis of the surgical bioprosthetic valve has been observed. The issue of whether to do an aortic valve repair or replacement is surgeon and center specific. Unfortunately, short-term or long-term data to compare the outcomes of these two approaches is limited and retrospective in nature. The advantages of a simple plication repair is that it requires a very short cross clamp time but leaves the aortic valve in the closed position and the patient is entirely dependent on LVAD support. In the aortic valve replacement requires a significant longer period of cardioplegic arrest of the heart but does allow for continued output across the aortic valve. The concerns about aortic valve replacement are that the long ischemic time may negatively impact right ventricular function and necessitate the need for a temporary right ventricular support device.

### 2.3 Diagnosis Post LVAD

Echocardiography can be diagnostic for aortic regurgitation and can track progression of the severity. However, due to continuous nature of regurgitation, aortic regurgitation can be underestimated by utilizing standard American Society of Echocardiography (ASE) guidelines for assessment. Grinstein and colleagues [13] proposed an approach that analyzes the pulse wave doppler profile of flow from the outflow cannula in the ascending aorta and correlated this hemodynamic based assessment with outcomes. However, this technique has not gained traction in clinical practice in part due to the challenges of imaging the outflow cannula on a surface echocardiogram. Transesophageal echocardiography can be a useful adjunct, especially if there are multiple and eccentric jets. Aortic root angiography is an underutilized diagnostic test in this population. This volumetric representation of regurgitant flow overcomes some of the pitfalls of doppler mapping of the vena contracta.

### 2.4 Intervention Post LVAD

Mild and moderate aortic regurgitation post LVAD can be managed with optimized medical management which is chiefly tight blood pressure regulation to avoid hypertension. Pump speed reduction has not been consistently demonstrated to decrease the risk and may further impair adequate LV unloading and lead to worsening HF symptoms [2]. Severe aortic regurgitation post LVAD often requires multi-disciplinary assessment. Decision regarding surgical or a percutaneous intervention requires careful evaluation of surgical risk, availability of transcatheter options and patient’s overall status. Surgical intervention can be in the form of AV closure (Dacron patch), AV repair (Park stitch), AV replacement, or heart transplant. However, redo sternotomy carries significant risks in LVAD patients including RV damage, dysfunction, and significant bleeding. Transcatheter therapies should also be considered alongside surgical approaches as part of an interdisciplinary approach to management. The European Associated for Cardio-Thoracic Surgery guidelines have strong preference for heart transplant when feasible (Class I) over open valve replacement/surgical closure (Class III) for moderate AR, with transcatheter AV replacement (Class IIa) and interventional closure of AV (Class IIb) receiving intermediate recommendations [14]. For severe AR, in patients who are not eligible for heart transplant, transcatheter aortic valve replacement (TAVR) is a Class IIa recommendation, and open valve replacement or closure and interventional closure have Class IIb recommendation.

### 2.5 Transcatheter AV Closure

**Fig. 3.**
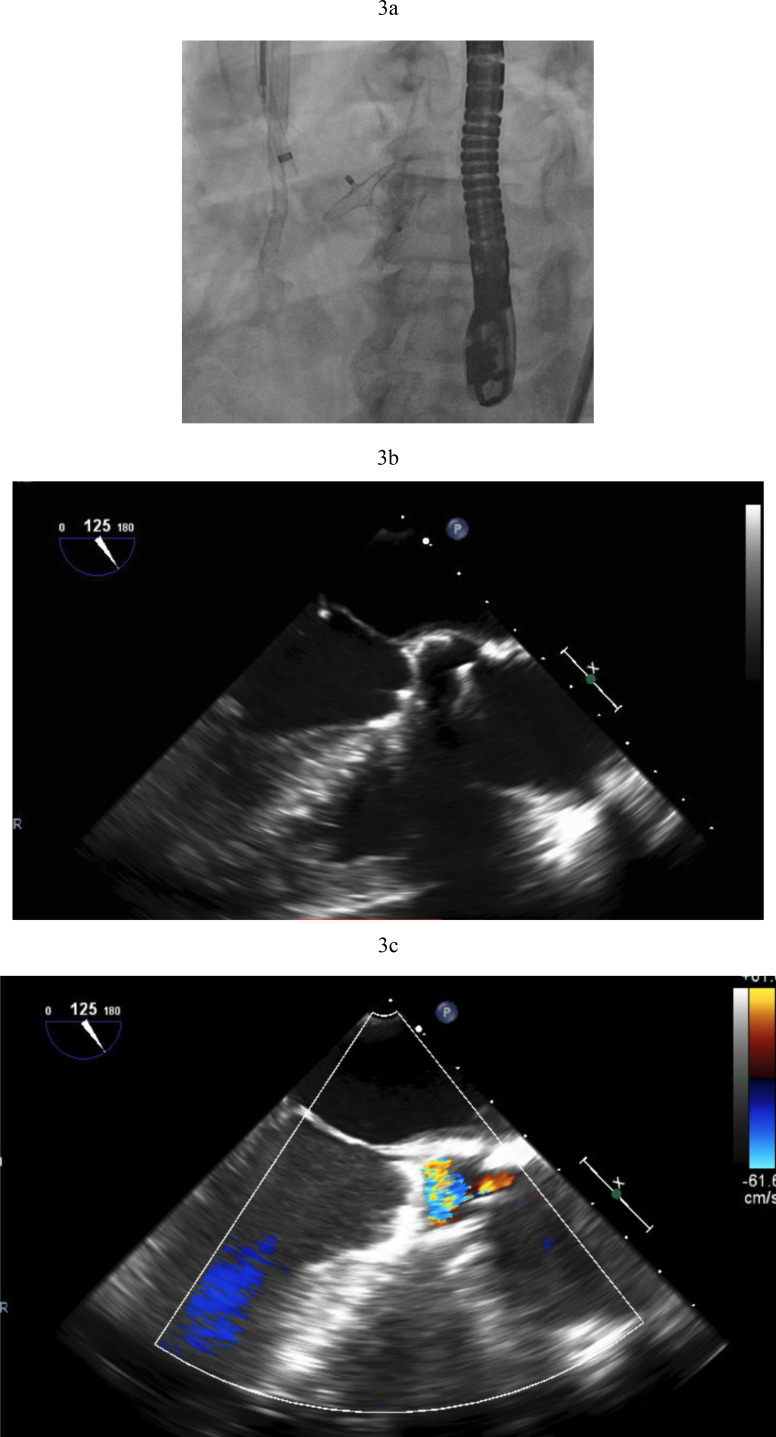
**Amplatzer closure of aortic valve**. (a) Amplatzer septal occluder across native aortic valve. (b) TEE showing the two discs of the Amplatzer device closing the aortic valve. (c) Aortic regurgitation resolved after Amplatzer closure. TEE, transesophageal echocardiogram.

Transcatheter AV closure was first reported in 2011 using Amplatzer Septal Occluders (Abbott Structural, Minneapolis, MN, USA). However, while this is a relatively uncomplicated procedure, long term outcomes are not favorable. Mortality in small case series is high with poor long term survival of 30% at 6 months [15]. Hemolysis is expected for a period of days after deployment until the device is fully endothelialized (Fig. 3a–c).

### 2.6 Transcatheter AV Replacement Post LVAD

TAVR is used for treating aortic stenosis and as a therapeutic option for aortic insufficiency resulting from failed bioprosthetic valves. TAVR has faced technical challenges in treating native aortic insufficiency, mainly because non-calcified leaflets do not allow for adequate device anchoring. The use of commercially available TAVR devices in native AR has been associated with suboptimal results including lower procedural success, higher need for a second heart valve and device embolization. Observational studies have shown more promising results with current generation TAVR devices in native aortic insufficiency despite the need to perform a valve-in-valve procedure up to 30% of cases in some series. In a meta-analysis of 12 studies that included 638 patients, Haddad *et al*. [16] compared the short-term outcomes of non-LVAD patients with pure native aortic regurgitation who underwent TAVR. The rate of device success was 92% with more recent iteration of TAVR valves which included Jena-Valve, Evolut R, J Valve and Sapien 3, with Jena-Valve representing the highest utilization in patients with native aortic regurgitation. Residual moderate or severe aortic regurgitation was 1% post TAVR.

**Fig. 4.**
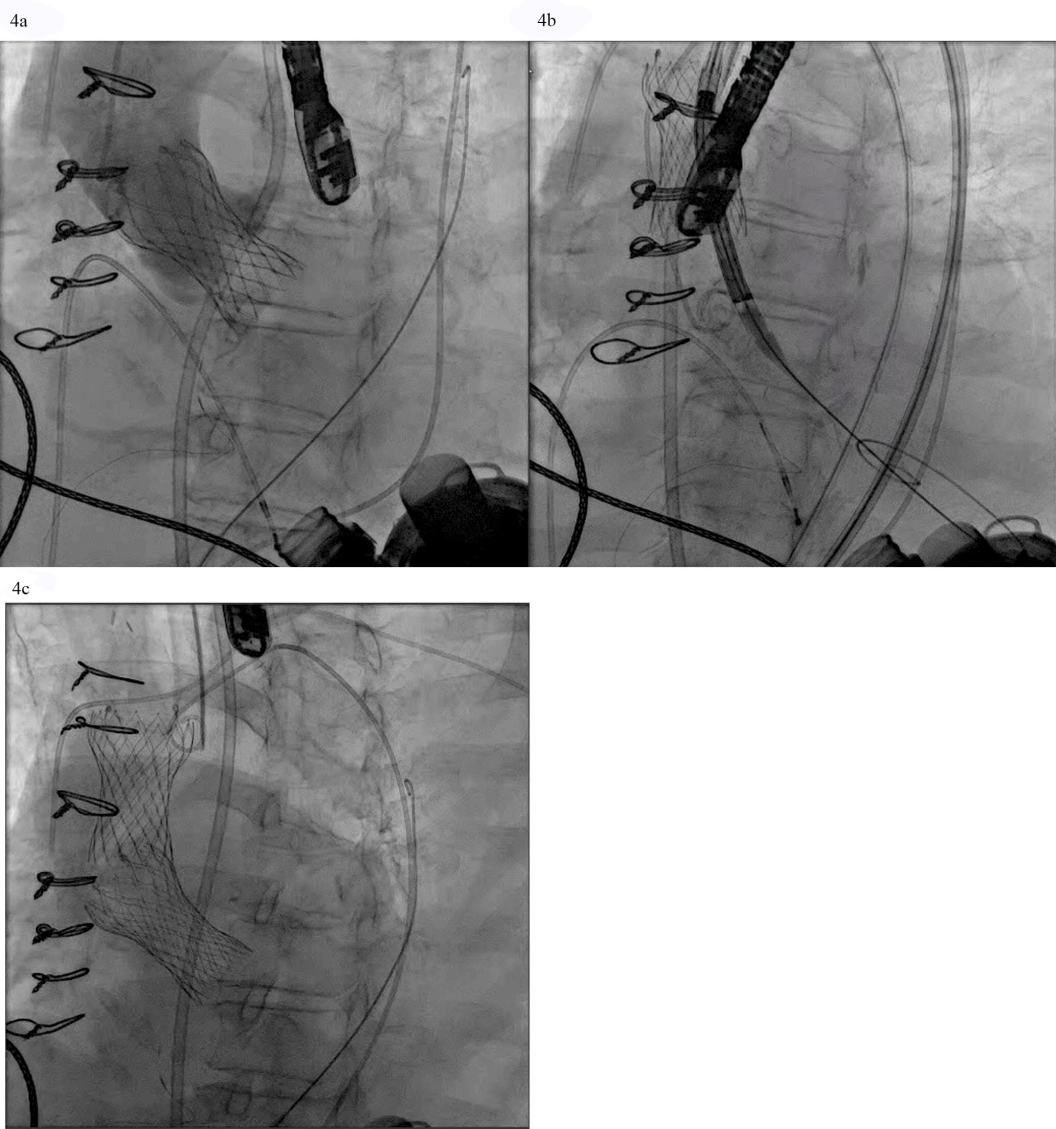
**AVR with a self-expanding valve (Medtronic) in LVAD complicated by migration**. (a) Medtronic Evolut valve deployed too deep in the ventricle. (b) Evolut valve embolized to the aorta when attempting to reposition the valve with a snare. (c) Second Evolut valve successfully placed across the aortic valve.

TAVR for AR in patients with LVADs poses additional challenges. The continuous pressure head in the aortic root from LVAD outflow cannula flow increases the risk of ventricular migration. If perivalvular regurgitation is present again the magnitude is magnified by the constant Ao-LV pressure gradient (Fig. 2). The absence of leaflet calcification leads to an increased risk of device misplacement or migration and paravalvular regurgitation from an incomplete seal (Fig. 4a,b). A valve-in-valve strategy in which a second valve is delivered to hold the first valve in place and prevent its migration has been deployed successfully (Fig. 4c). To minimize this risk, LVAD flows should be temporarily decreased during valve deployment. A single center experience which utilized predominantly the self-expanding Evolut TAVR platform reported that 36% of the procedures required a second valve [17]. The French registry of TAVR for native aortic regurgitation noted an 8.8% rate of second valve implantation that portends a worse clinical outcome [18].

**Fig. 5.**
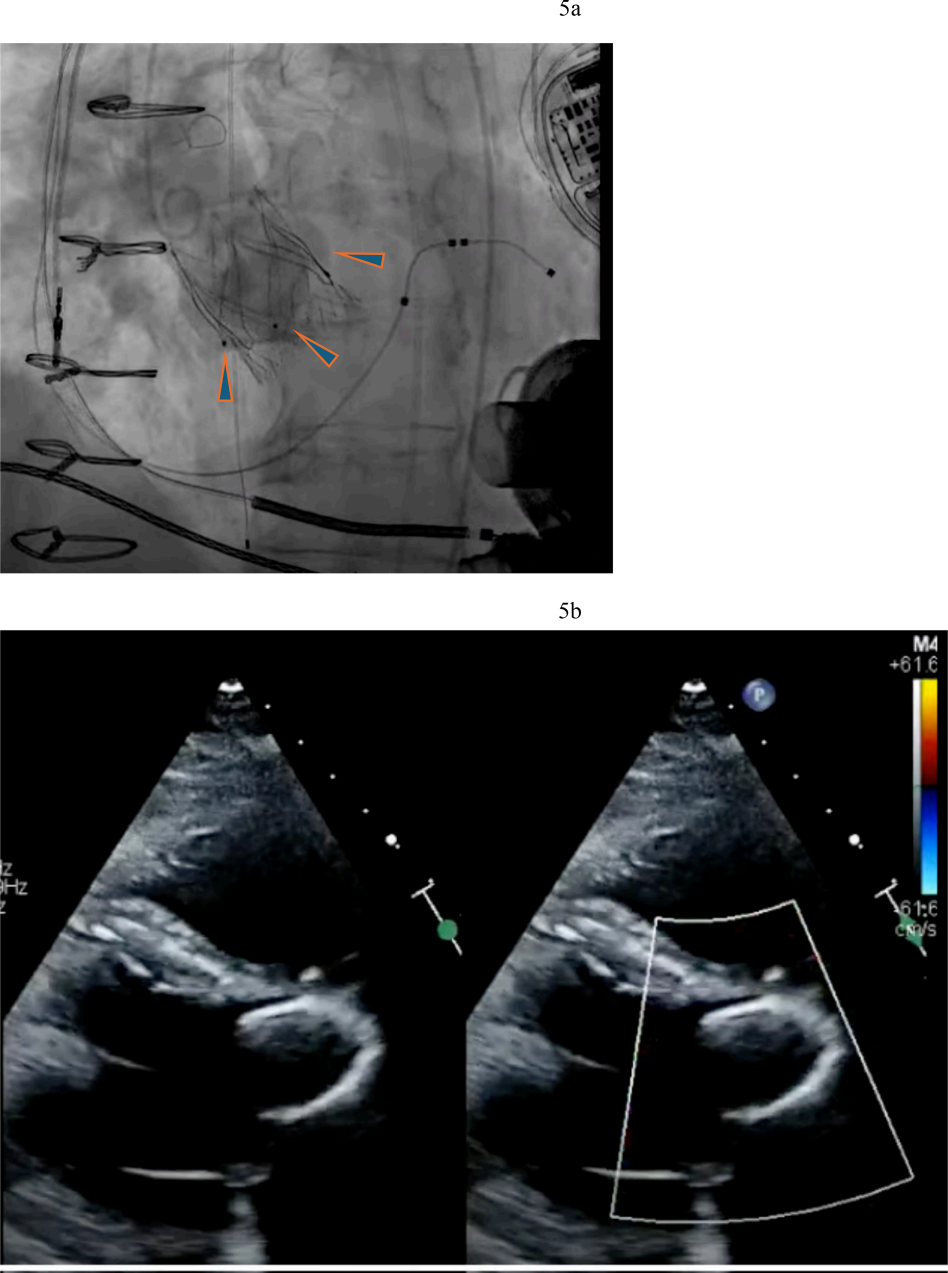
**Jena-Valve in LVAD patient with severe AR**. (a) Jenavalve 27 mm with three locators (arrowheads) anchored to the aortic valve. (b) Resolution of AR after Jenavalve implantation.

The emergence of dedicated devices for aortic regurgitation which rely on leaflet capture for device fixation (JenaValve and J-Valve) has the potential to address this unmet clinical need. The JenaValve (JenaValve Technology, Inc., Irvine, California, USA) is the only currently available device with CE mark for the treatment of aortic regurgitation. It is currently under investigation in the USA. The JenaValve is a porcine pericardial valve on a self-expanding nitinol frame made of three integrated arms called locators. The locators align the valve with the native aortic valve native aortic valve anatomy and clip onto the native leaflets providing fixation independent of valve calcification. Ranard *et al*. [19] recently reported the first use of the JenaValve to address severe aortic regurgitation in a LVAD patient. We have utilized the Jena-Valve platform in LVAD patients with technical success and excellent clinical results (Fig. 5a,b).

**Fig. 6.**
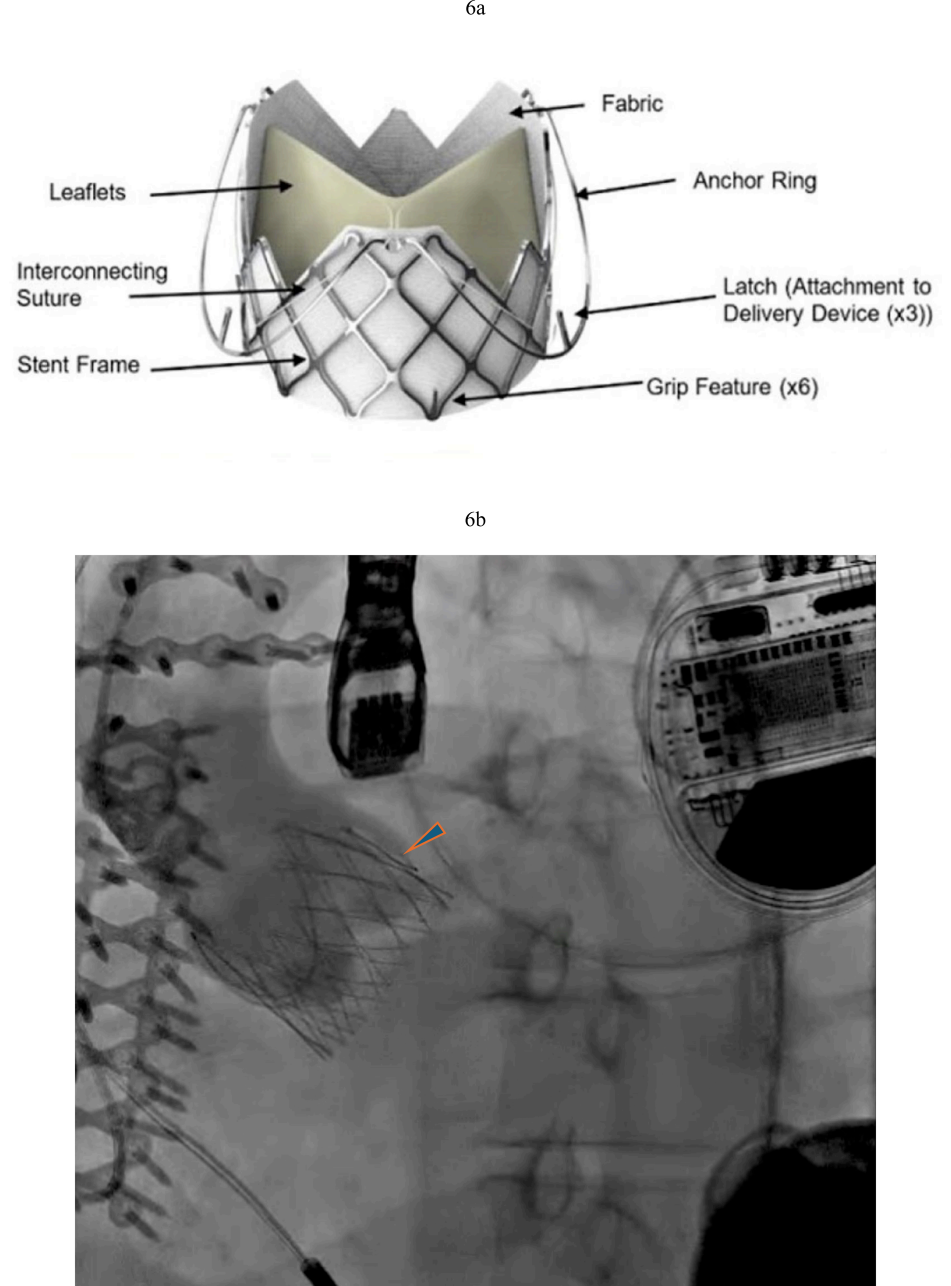
**J Valve TAVR in LVAD patient with severe AR**. (a) J-Valve with three anchor rings that secure in the aortic cusps. (b) J Valve implantation with resolution of AR (arrowhead pointing to anchor ring).

J-Valve (JC Medical Technologies, Irvine, CA, USA) is a bovine pericardial valve on a self-expanding nitinol frame with 3 U-shaped anchor rings that facilitate alignment with the native aortic valve anatomy and anchor the valve frame to the native leaflets. Garcia *et al*. [20] have demonstrated successful use of the J-Valve system in a patient with severe aortic regurgitation associated with a LVAD with excellent results (Fig. 6). Both valve platforms plan to enroll LVAD patients in a dedicated nested registry as part of the regulatory approval.

Off- label use of TAVR devices designed for Aortic stenosis – Evolut (Medtronic Inc., Minneapolis, MN, USA) and Ssapien 3 (Edwards Lifesciences Inc., Irvine, California, USA) systems has been performed with variable results. There is insufficient data on which of the two commercially available systems is better suited for this. Evolut valve has the advantage of ascending aortic fixation due to large outflow diameter, while the S3 and its balloon-expandable deployment requires deliberate oversizing (>20%) to improve anchoring in the annulus and leaflets. In some cases, both valves are required to achieve fixation and improve procedural success. The Evolut frame fixation within the aorta serves as a scaffold to anchor the Sapien 3 in the absence of annular calcifications. However, due to the prohibitive costs associated with the use of two prostheses, this technique is not generalizable. The ability to recapture and reposition the Evolut valve at up to 80% deployment helps with improving deployment success. During positioning the valve, the LVAD pump speed is decreased as tolerated. After completing deployment, we maintain reduced speed and pacing for a longer run to reduce the risk of valve migration and embolization. We return the LVAD to its nominal speed by a slow ramp with echocardiographic visualization. Ventricular migration is more frequent than embolization with Evolut valve. Aortic embolization can occur during attempts to reposition the valve using snares. If the Evolut valve migrates into a suboptimal position our strategy is to deploy a balloon-expandable valve (Sapien 3) inside the valve frame. Following TAVR the procedure in an LVAD patient meticulous management of post-operative anticoagulation with strict adherence to an international normalized ratio (INR) goal >2.0 is necessary to avoid valve leaflet thrombosis.

**Fig. 7.**
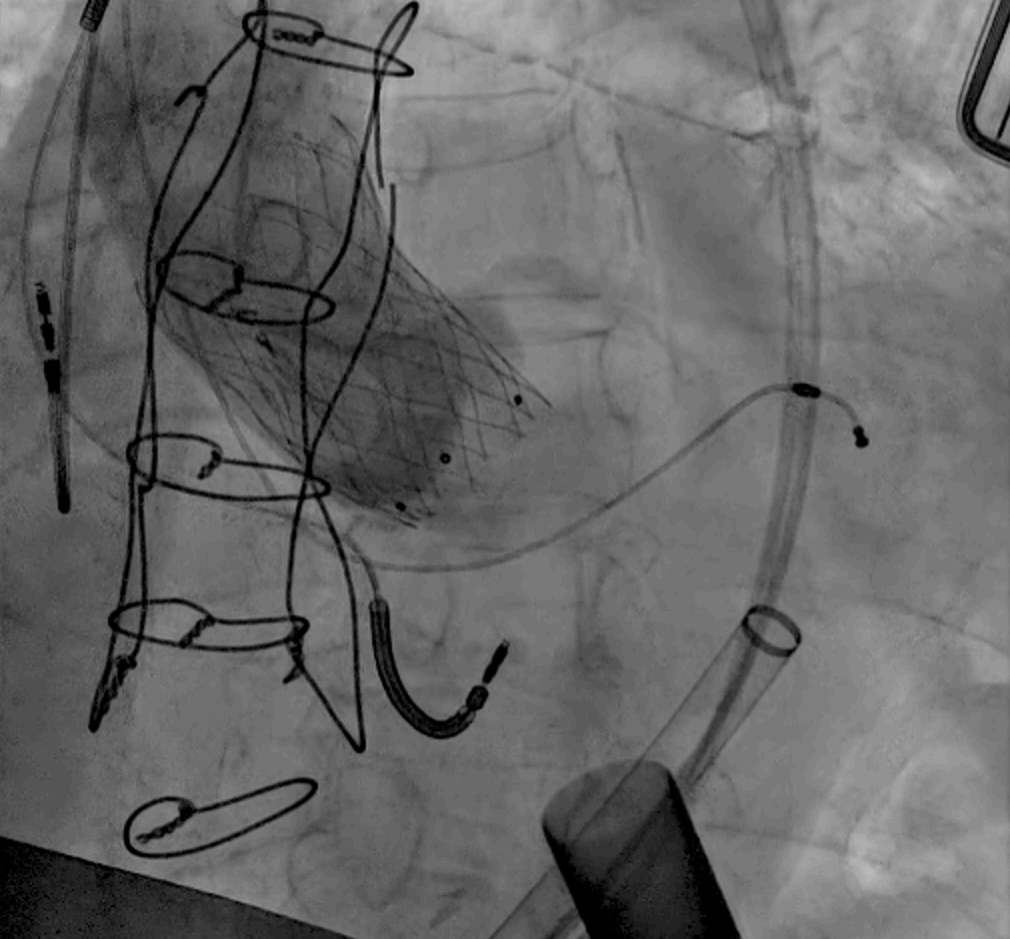
**Abbott Navitor TAVR in LVAD patient**. Abbott Navitor valve with large outflow diameter and anchoring in the ascending aorta for AR.

The newly approved Navitor valve (Abbott Laboratories, Minneapolis, MN, USA) has a larger outflow diameter than the Evolut platform which should improve aortic anchoring and similar to the Evolut platform can be deployed at up to 80% expansion to assess valve function, recaptured and repositioned as needed. We have successfully deployed this valve in two LVAD patients with severe aortic regurgitation and no evidence of calcium at the aortic root or valve leaflets (Fig. 7).

## 3. Mitral Valve Disease

### 3.1 Considerations at Time of Implant

Mitral valve replacement or repair at time of LVAD implant is not common, and there is no consensus guideline or best practice [21]. Mitral valve dysfunction — either native or prosthetic — is common at time of LVAD implantation. The most typical scenario is the presence of significant functional mitral regurgitation, which is at least moderate or worse in ~60% patients at time of LVAD [22]. The unloading that occurs with an LVAD usually reduces the mitral regurgitation to clinically unimportant levels so that the surgeon can often avoid mitral intervention at time of LVAD placement. However, there are LVAD patients that have residual mitral regurgitation following implantation. In an INTERMACS analysis, Jain and colleagues [3] showed no evidence of increased mortality risk in those that had moderate or worse residual MR; however residual MR was associated with increased incidence of both renal failure and right ventricular failure. Significant MR post LVAD is associated with RV dysfunction. It is difficult to determine if the RV dysfunction is the result of MR (due to pulmonary hypertension) or the cause of MR (inadequate LVAD pump speed due to RV dysfunction). In patients with mitral stenosis, either native or iatrogenic (transcatheter edge-to-edge repairs (TEER), prosthetic valve dysfunction), there is broad consensus to address the lesion at time of LVAD, because with increased left sided flow, degree of mitral stenosis (MS) will be more pronounced after LVAD implantation. Our general practice has not been to address the mitral valve (MV) for MR, but for obstruction and significant stenosis of the native valve or prosthethic valve. If there is normal functional bioprosthetic or mechanical mitral valve then it is left in place at time of LVAD implant.

### 3.2 Mitral Regurgitation Post Implant

Post LVAD significant residual MR results in the clinical sequelae of pulmonary congestion, persistently elevated left sided filling pressure, pulmonary hypertension, and severe LV dilation. The initial approach is to optimize the unloading of the left ventricle with pump speed adjustments, diuresis and afterload agents. In most cases the degree of mitral regurgitation can improve. The interaction between co-existent right ventricular dysfunction and mitral regurgitation must be characterized to determine whether pump speed interventions or valve interventions will lead to improve hemodynamics and patient’s clinical status. Echocardiography provides parameters of function and volume by 3D analysis, as well as qualitative assessment of the relative position of the interatrial and interventricular septum towards the right versus the left side. Right heart catheterization provides parameters of filling pressures, right atrial to pulmonary capillary wedge ratio and pulmonary artery pulsatility index.

In instances in which residual MR does not improve percutaneous therapies such as mitral valve transcatheter edge to edge repair (M-TEER) [23] and transcatheter mitral valve replacement (TMVR) [24] may be considered. Mitral regurgitation has been successfully treated with mitral TEER in patients with severe LV dysfunction [25]. COAPT trial showed reduction in mortality and HF admissions. MITRA-FR trial on the other hand did not show reduction in mortality or HF in patients with secondary MR. The severity of MR and LV size between the trials accounted for the differing results. RESHAPE-HF2 trial showed reduction in HF admissions in patients with moderate to severe MR but smaller LV chamber dimensions compared to patients enrolled in MITRA-FR trial. MR in patients with LVAD is well tolerated however in some patients disproportionately severe MR may need to be addressed with M-TEER especially when optimization of guideline directed therapy and LVAD support does not improve symptoms. In patients with degenerated mitral valve bioprosthesis, transcatheter mitral valve replacement (valve-in valve or valve-in-ring) is minimally invasive compared to redo surgery and significantly lower risk. Heart team discussion prior to proceeding with transcatheter mitral valve therapy is highly recommended. Both techniques can improve mitral regurgitation resulting in improved left atrial pressures and a subsequent reduction in pulmonary vascular resistance that can improve right ventricular function. The iatrogenic atrial septal defect post procedure should be addressed in the setting of right ventricular dysfunction and/or tricuspid regurgitation as to avoid significant hypoxemia due to right to left shunting. For TMVR procedures in an LVAD patient meticulous management of post-operative anticoagulation with strict adherence to an INR goal >2.0 is necessary to avoid valve leaflet thrombosis.

## 4. Tricuspid Regurgitation

### 4.1 Considerations at Time of Implant

Given the preponderance of RV dysfunction in advanced HF patients, particularly after LVAD implantation, coexistence of significant tricuspid regurgitation is a clinical challenge. Pulmonary hypertension and presence of transvalvular pacemaker/defibrillator leads likely contribute to TR as well. The prevalence of significant tricuspid regurgitation (moderate or worse) is approximately 30–40% [26]. The question of tricuspid valve (TV) repair at time of LVAD implant is controversial, without clear guidelines for management. In most cases tricuspid regurgitation will improve over time. However, there will be a cohort of patients that develop worsening TR or the remodeling process to improve tricuspid regurgitation does not occur fast enough such that the hazard of right ventricular dysfunction is exacerbated. Preoperative transesophageal echocardiogram may not accurately predict the degree of tricuspid regurgitation seen after LVAD implant. Post LVAD TR may worsen or develop de novo due to tricuspid annular enlargement due to septal shift to the left, due to increases in pulmonary vascular resistance (PVR) that occur with hypoxia and hypercapnia, as well as fluid shifts that overload the right ventricle. De novo tricuspid regurgitation occurs approximately 10% of the time [4]. Significant tricuspid regurgitation in the post-operative time period can cause worsening right ventricular failure may lead to poor outcomes, including requirement for right ventricular assist devices (RVADs), renal dysfunction and/or death [27].

A small randomized controlled trial was completed in patients undergoing LVAD with preoperative moderate or severe tricuspid regurgitation [28]. At six months there was no difference in right heart failure — however by the trial’s definition right heart failure was very sensitive with 46.9% incidence in the TV surgery arm versus 50% in the no TV surgery arm. Interestingly in this small study there was a trend toward improved survival and fewer RVADs in the group that received TV surgery. There is no data to suggest that TV repair improves long term outcomes. However, driven by a desire to remove as many variables as possible for RV management in the immediate post-operative period, our practice has been to repair the TV if the baseline transesophageal echocardiogram (TEE) shows moderate or greater TV regurgitation of if TV annular diameter at end systole is 4 cm or greater.

### 4.2 Tricuspid Regurgitation Post LVAD

As structural heart operators gain experience in the novel transcatheter therapies for tricuspid regurgitation we expect more utilization and application to patients with LVADs. Optimization of fluid status, right ventricular afterload reduction, and pump speed adjustments to balance the interventricular septum are the first steps in management of the LVAD patient with significant tricuspid regurgitation. If severe tricuspid regurgitation persists and results in symptoms and HF hospitalizations for heart failure then evaluation by heart team for transcatheter therapies is recommended. Anatomical and clinical factors are important to consider when selecting between transcatheter tricuspid edge to edge repair (T-TEER) or valve replacement (TTVR). These include RV function, leaflet coaptation gaps (>10 mm unfavorable for TEER), the presence of pacemakers or defibrillator leads and bleeding risk. Due to more complete elimination of TR, TTVR can lead to acute RV dysfunction due to increased afterload. Patients with pre-existent severe RV dysfunction and/or severe pulmonary hypertension (pulmonary artery systolic pressure (PASP) ≥70 mmHg) have not been studied in pivotal trials but is generally accepted that these patients are not ideal candidates for TTVR. Similarly, TTVR can jail cardiac implantable electronic device (CIED) leads, which can lead to malfunction or inability to remove the leads in case of infection. A careful evaluation of risks and benefits of jailing versus extracting CIED leads is an important step prior to TTVR implantation. This discussion should be informed by an electrophysiologist experienced in lead extraction.

**Fig. 8.**
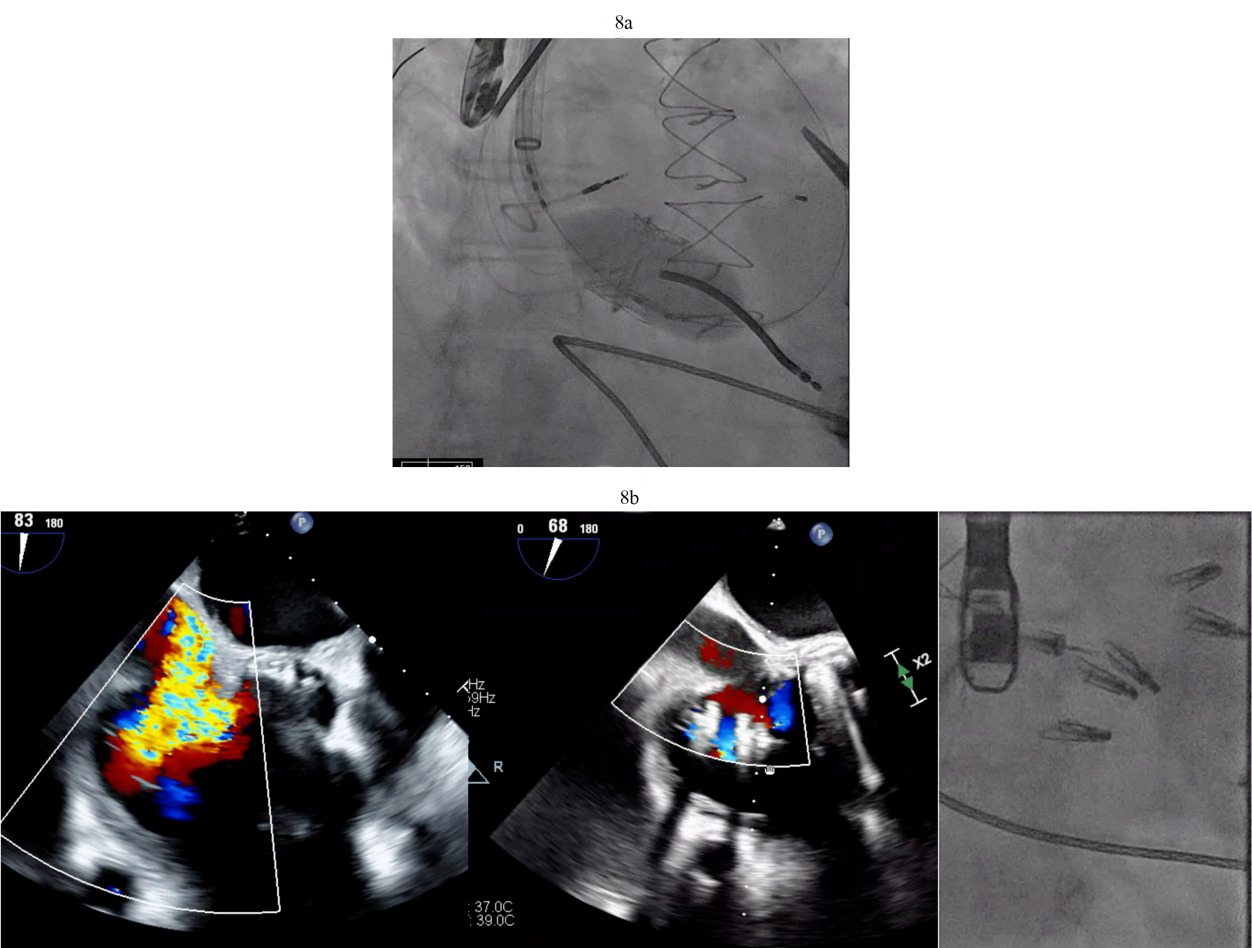
**Tricuspid valve valve-in-ring TTVR in LVAD patient**. (a) Sapien 3 valve being deployed in a TV annuloplasty band for treatment of severe TR via trans jugular approach. (b) T-TEER with Triclip. Torrential to mild TR after three Triclips. (Two previously placed Mitraclips are also visible on the fluoroscopy image). T-TEER, tricuspid transcatheter edge to edge repair; TV, tricuspid valve; TR, tricuspid regurgitation.

In LVAD patients that have had prior ring annuloplasty repairs and severe tricuspid regurgitation, transcatheter TV replacement is an option utilizing commercially available TAVR valves in a surgical ring approach. Our group successfully performed valve-in-ring TTVR in an LVAD patient with severe TR refractory to medical treatment (Fig. 8a). T-TEER in an LVAD patient (Fig. 8b) has been performed by our group and others [29]. As discussed above CIED leads are present in a large portion of LVAD patients and this approach may not be as successful given the anatomical challenges including large coaptation gaps (>10 mm), and imaging artifacts created by CIED leads.

## 5. Patent Foramen Ovale and Atrial Septal Defect

### 5.1 Considerations at Time of Implant

The existence of a defect in the interatrial septum can be problematic in an LVAD patient. When left atrial pressure is chronically higher than the right atrium, significant left to right shunting can further volume stress the RV. When LV unloading is brisk with LVAD, there can be right to left shunting that leads to hypoxia. An unaddressed septal defect (atrial septal defect (ASD), patent foramen ovale (PFO), iatrogenic defect) represents yet another variable to post-operative management of the patient; thus, when the defect is recognized preoperatively surgical closure is recommended. The PFO is closed primarily with running sutures with bicaval cannulation. This procedure can be performed without aortic cross clamp and is best performed before the creation of the outflow graft anastomosis, as the outflow can preclude easy access to the right atrium once it is secured in place. In situations where a significant shunt is discovered after the LVAD with associated clinical challenges as noted above, transcatheter intervention can be performed. The 2023 ISHLT guidelines [10] recommend that (1) Preoperative assessment of the presence of interatrial communication should be performed using TEE and (2) Closure of a significant interatrial shunt should be performed.

### 5.2 Right to Left Shunting Post LVAD

**Fig. 9.**
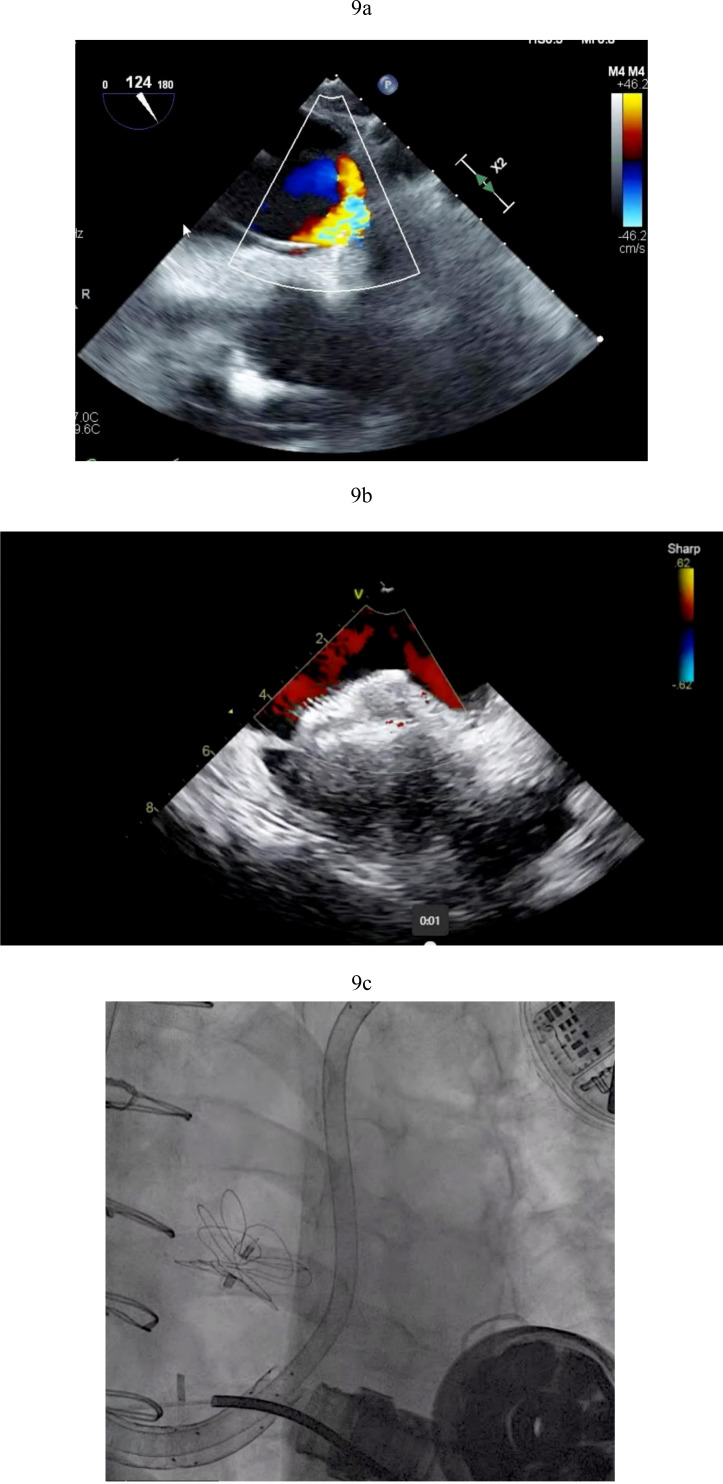
**PFO closure post LVAD**. (a) Large right to left shunt in a patient with PFO and RV failure. (b) Amplatzer closure of PFO in LVAD patient. (c) Successful closure of PFO with Gore 32 mm ASD occluder. Patient on RVAD support due to worsening RV failure in the settting of hypoxemia. RV, right ventricular; RVAD, right ventricular assist device.

The determination of what is significant is driven by two factors. The size of the defect certainly plays a role but more importantly one needs to consider that RV dysfunction and elevated right -sided filling pressures may occur post LVAD in the immediate post operative state. PFOs can become very problematic in circumstances of significant RV failure post LVAD. As right atrial pressures rises, right to left shunting increases (Fig. 9a). This shunting can result in hypoxemia that worsens pulmonary vascular resistance and leads to worsening right ventricular function with further increases in right atrial pressures. This vicious cycle can quickly derail a stable post-operative course. Percutaneous PFO closure can effectively stop right to left shunting and ameliorate symptoms (Fig. 9b). In situations where right ventricular hemodynamics are tenuous, compromising LVAD preload and flows and jeopardizing end organ function, RVADs may be required to stabilize the patient’s condition prior to PFO closure (Fig. 9c).

## 6. LVAD Outflow Cannula Obstruction

Obstruction to flow through the LVAD outflow graft (OFG) is an increasingly recognized complication that can lead to pump dysfunction and worsening heart failure. Clinical presentation can vary depending on the degree of obstruction and degree of native LV contractility. Patient symptoms can range from exertional dyspnea, fatigue and dizziness to as dramatic as cardiogenic shock. The most common presentation is with low LVAD pump flows. The incidence has been estimated by one case series at 3% per year [30]. There are multiple potential mechanisms, but two are predominant — (1) Kinking/Twisting and (2) Extrinsic compression [31]. In the early post-operative period OFG is often secondary to kinking or twisting. Detection is made by contrast computed tomography (CT) angiogram with cardiac cycle gating to image the OFG. This complication requires surgical intervention to correct. This can be achieved by shortening or repositioning. The mechanism that is more commonly seen, in the chronic setting, and has an insidious presentation is OFG obstruction due to external compression. The location is typically at the proximal outflow cannula in the region of the bend relief, where a piece of polytetrafluoroethylene (PTFE) is used to wrap the OFG at time of implant to protect from twisting and injury if a redo-sternotomy is required. It appears that a thick, proteinaceous exudate from the OFG can get trapped between the OFG and the PTFE wrap leading to external compression. Often one needs a high index of clinical suspicion for this type of OFG obstruction does not usually result in hemolysis on routine bloodwork nor is detected by echocardiography. Cardiac CT angiogram is the diagnostic test of choice though measurement of gradients within the OFG in the catheterization laboratory can help determine its significance. This complication led to the Food and Drug Administration (FDA) announcing a recall for both the HM2 and HM3 devices from Abbott in 2024. The time course of this process has not been well characterized but in our experience can present in the course of at least year out from time of implant to the point that it becomes clinically significant.

**Fig. 10.**
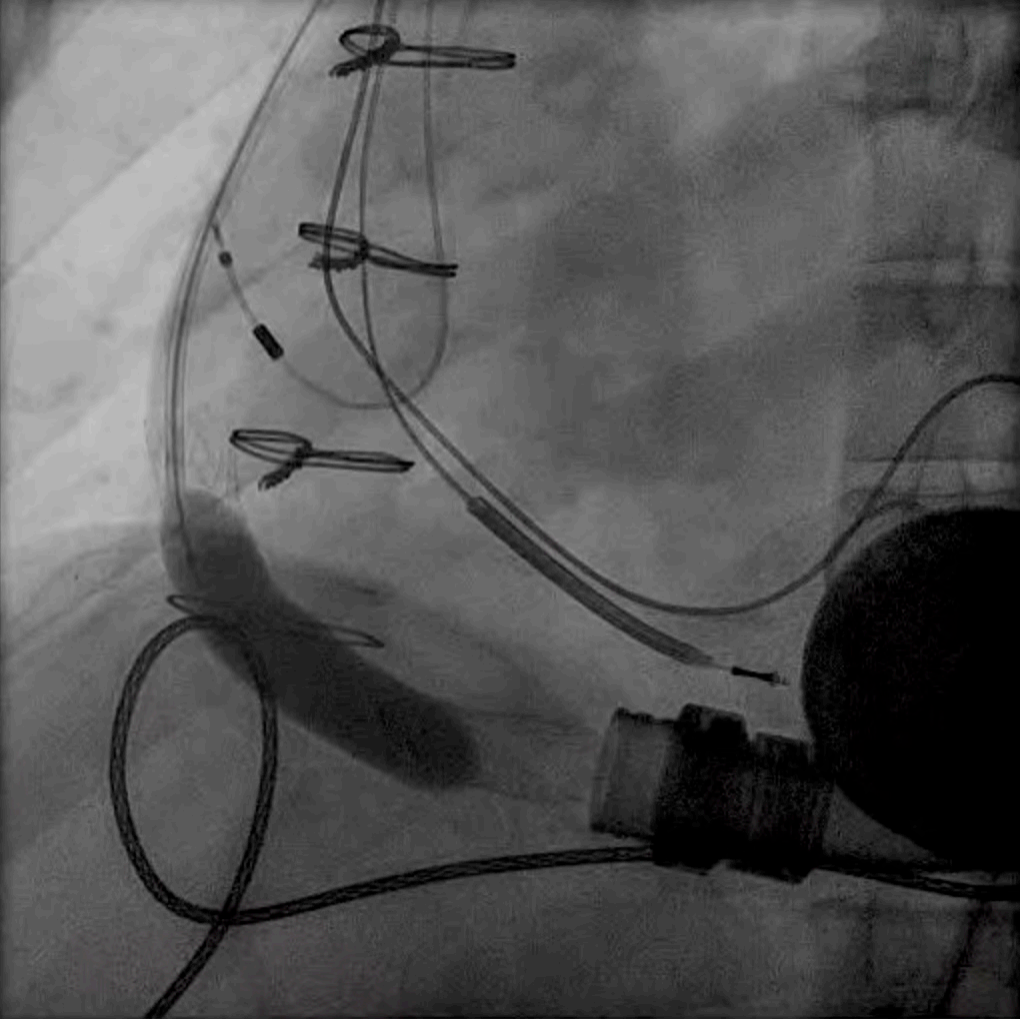
**Outflow cannula obstruction intervention**. Outflow cannula obstruction treated with a large self-expanding vascular stent and dilated with a 10 mm balloon.

For OFG narrowing due to external compression, percutaneous interventions can dramatically restore LVAD pump flows and improve patient symptoms. In the cath lab, angiography and concomitant measurement of pressure gradients can help identify the location and extent of the outflow cannula narrowing. Pressure gradients are often not dramatically elevated but even a modest gradient over a longer length of narrowing can create significant resistance. We have treated these situations using balloon angioplasty with excellent outcomes (Fig. 10). The decision to place a stent depends on whether balloon angioplasty alone leads to adequate relief, and if the patient can tolerate a period of treatment with P2Y12 inhibitors.

## 7. LVAD Pump Thrombosis

Pump thrombosis is a potentially catastrophic complication of LVAD therapy. Presentation can vary from asymptomatic hemolysis to dramatic pump stoppage and cardiogenic shock. Diagnosis is made by the presence of hemolysis in bloodwork and evidence of pump dysfunction by echocardiography. In the Endurance trial that compared the first generation continuous flow LVAD HM2 with the second generation continuous flow LVAD HeartWare ventricular assist device (HVAD), the incidence of pump exchange due to pump thrombosis at 2 years was 10.7% for HM2 device and 6.7% for the HVAD device [32]. Fortunately, this dreaded complication is infrequently seen with the most current, third generation LVAD, the HM3. However, there are still many patients with implanted HM2 and HVAD who may present with some degree of pump thrombosis, and clinicians have explored whether percutaneous delivery of thrombolytics can salvage a pump and therefore prevent a high risk pump exchange surgery or urgent listing for heart transplantation.

**Fig. 11.**
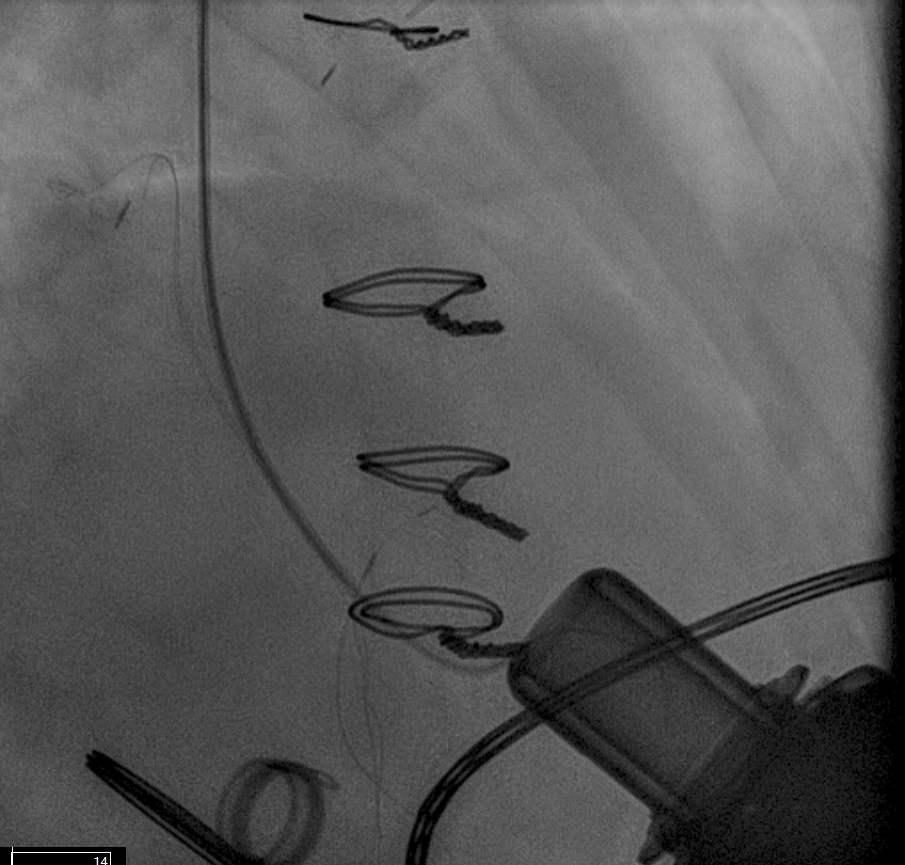
**Catheter based thrombolytics in pump thrombosis**. Pigtail catheter placed in the LV close to the LVAD inflow and thrombolytic infusion over a period of 24 hours. LV, left ventricle.

Percutaneous delivery of thrombolytics can be achieved by retrograde placement of a pigtail catheter across the aortic valve, locating the tip within the inflow cannula of the LVAD (Fig. 11). Patients are then monitored in the ICU and thrombolytic therapy can be delivered over a time period of 48–72 hours. Pump thrombosis of the HVAD tends to be more responsive to thrombolytic therapy that thrombosis in HM2, which is likely in part due the mechanism of pump thrombosis. Jorde and colleagues [33] demonstrated that log file analysis depicted a time course of clot formation within the pump and that in cases where there was a gradual buildup this responded better to thrombolytics as opposed to a sudden increase in power likely due to an ingested, old clot that would not be expected to dissolve with thrombolytic agents. In their series of HVAD pump thrombosis the success rate of thrombolytics was 57%. A meta-analysis of multiple reports of thrombolytics demonstrated a success rate of 65% with an incidence of major bleeding at 29% [34]. If the pump has been salvaged with percutaneous thrombolytics then the INR goal for anticoagulation is typically increased to 2.5 to 3.5 and often the anti-platelet inhibitor is escalated, for example substituting clopidogrel in place of aspirin.

## 8. LVAD Decommissioning

Recovery of LV function to the point that the patient no longer requires mechanical circulatory support from the LVAD occurs in a small population of patients. The incidence of patients experiencing recovery to the point of deactivation from national registries is 1–2% [35]. There is a higher percentage of patients that experience some degree of recovery, however not to the point of liberating them from their device. There is active research in working towards optimizing recovery and increasing this outcome. Patients that are considered responders and have adequate recovery to consider LVAD deactivation when they have an LVEF ≥45 and LVEDD <6.0 cm. The decision to proceed comes after “turn-down” studies in which the pump speed is decreased to levels at which there is approximated no flow through the pump (HM2 6000 rpm, HVAD 1800 rpm and HM3 4000 rpm), with no evidence of decompensation on echocardiography and right heart catheterization. The patient should be fully anticoagulated for this test. Prior to deactivation it is important to discuss with the patient the risk of relapse of their heart failure symptoms and how this might impact the quality of life and survival. A single center experience demonstrated the survival free of re-implant LVAD, heart transplant and death is 90% at 1 year and 77% at 3 years [36]. In addition to the hemodynamic parameters obtained from turned down studies, psychologic and social factors are considered in the context of a multidisciplinary team discussion prior to proceeding with LVAD deactivation.

**Fig. 12.**
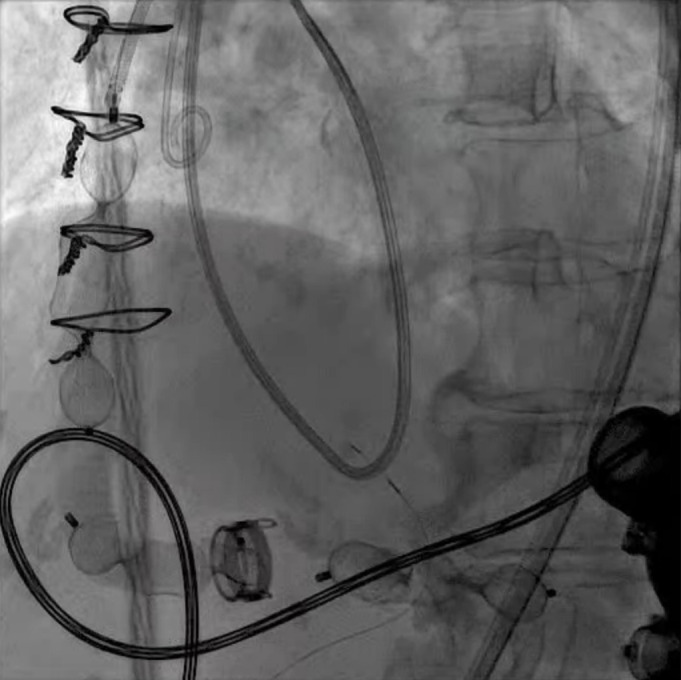
**LVAD decommissioning**. Outflow cannula occlusion with a series of three large Amplatzer vascular plug 2 (AVP2) from the proximal end to the distal end of the cannula.

LVAD deactivation can be achieved by surgical explant via median sternotomy or via a hybrid surgical/percutaneous approach described as decommissioning [37]. Complete explant is recommended in younger patients and those with infected drivelines and/or pump. Decommissioning implies retention of the pump within the body. The driveline is cut and buried subcutaneously. The outflow cannula must be occluded to prevent closed loop regurgitant flow from the aorta into the left ventricle, typically with endovascular occlusive device/s at the OFG-ascending aorta anastomosis (Fig. 12). Surgical decommissioning can be achieved minimally invasively and without cardiopulmonary bypass.

## 9. Conclusion

Although we provide comprehensive guidance on the management of complex structural abnormalities in LVAD patients with the aim to improve the care and outcomes of this patient population, evidence in support for our therapeutic strategies rests mostly on retrospective studies and case series, and individual treatment options rely heavily on the collective expertise of our multi-disciplinary heart team. Larger-scale prospective studies are needed to validate the long-term safety and efficacy of different devices. Valvular lesions both at the time of implant as well as post LVAD add complexity to the care of these patients with advanced heart failure. The natural course of these lesions impact decisions made in the operating room. Aortic regurgitation has a high incidence of development and progression post LVAD due the unique physiologic forces on the mostly closed aortic valve. Novel TAVR therapies are well suited to address aortic regurgitation post LVAD. We are hopeful that longer-term data on the use of TAVR in patients with LVAD’s will be forthcoming from registries established by the TAVR device companies. Both Jena and J-Valve will have single-arm LVAD registries running in parallel to their pivotal clinical trial (Clinical trials.Gov ID NCT06594705), the JenaValve ALIGN-AR LVAD Registry (JENA-VAD). In general, both mitral regurgitation and tricuspid regurgitation improve after LVAD implantation. However, in scenarios where significant MR and/or TR do not improve or newly develop, the clinical course of the LVAD patient is challenged by heart failure symptoms including impaired activity tolerance and pulmonary congestion. Right ventricular dysfunction co-exists in both these scenarios making the decision regarding valve intervention challenging to predict whether addressing the valvular regurgitation with percutaneous therapies is enough to improve the trajectory of the LVAD patient who presents with heart failure. Circumstances unique to the LVAD, outflow cannula obstruction, pump thrombosis and decommissioning can be addressed with techniques and devices developed in the structural heart and interventional cardiology arenas. The collaboration by cardiologist and cardiac surgeons with diverse skill sets and hemodynamic understanding creates a synergistic environment to solve some of the most complex problems in cardiovascular medicine. This review provides a systematic comparison of the advantages and disadvantages of various interventional strategies for LVAD patients, offering new perspectives for clinical decision-making.
